# Enhancing striatal acetylcholine facilitates dopamine release and striatal output in parkinsonian mice

**DOI:** 10.1186/s13578-024-01328-z

**Published:** 2024-12-03

**Authors:** Hongxia Li, Ziluo Chen, Yuyan Tan, Huoqing Luo, Chen Lu, Chao Gao, Xin Shen, Fang Cai, Ji Hu, Shengdi Chen

**Affiliations:** 1 Department of Neurology & Institute of Neurology, Ruijin Hospital, Shanghai Jiao Tong University School of Medicine, Shanghai, China; 2 Lab for Translational Research of Neurodegenerative Diseases, Institute of Immunochemistry, ShanghaiTech University, Shanghai, China; 3 School of Life Science and Technology, ShanghaiTech University, Shanghai, China

**Keywords:** Acetylcholine, Dopamine, Striatum, Parkinson’s disease, In vivo

## Abstract

**Background:**

L-DOPA has been considered the first-line therapy for treating Parkinson’s disease (PD) via restoring striatal dopamine (DA) to normalize the activity of local spiny projection neurons (SPNs) in the direct (dSPNs) pathway and the indirect (iSPNs) pathway. While the changes in striatal acetylcholine (ACh) induced by increasing DA have been extensively discussed, their validity remains controversial. Inhibition of striatal cholinergic signaling attenuates PD motor deficits. Interestingly, enhancing striatal ACh triggers local DA release, suggesting the pro-kinetic effects of ACh in movement control. Here, we investigated the in-vivo dynamics of ACh in the dorsolateral striatum (DLS) of the 6-OHDA-lesioned mouse model after L-DOPA administration, as well as its underlying mechanism, and to explore its modulatory role and mechanism in parkinsonian symptoms.

**Results:**

Using in vivo fiber photometry recordings with genetically encoded fluorescent DA or ACh indicator, we found L-DOPA selectively decreased DLS ACh levels in parkinsonian conditions. DA inhibited ACh release via dopamine D2 receptors and dSPNs-mediated activation of type-A γ-aminobutyric acid receptors on cholinergic interneurons. Restoring DLS ACh levels during L-DOPA treatment induced additional DA release by activating nicotinic acetylcholine receptors, thereby promoting the activity of dSPNs and iSPNs. Enhancing DLS ACh facilitated L-DOPA-induced turning behavior but not dyskinesia in parkinsonian mice.

**Conclusions:**

Our results demonstrated that enhancing striatal ACh facilitated the effect of L-DOPA by modulating DA tone. It may challenge the classical hypothesis of a purely competitive interaction between dopaminergic and cholinergic neuromodulation in improving PD motor deficits. Modulating ACh levels within the dopaminergic system may improve striatal DA availability in PD patients.

**Supplementary Information:**

The online version contains supplementary material available at 10.1186/s13578-024-01328-z.

## Background

Parkinson’s disease (PD) is the second most common neurodegenerative disease, characterized by the degeneration of dopaminergic neurons in the substantia nigra pars compacta (SNc), inducing dopaminergic denervation of the striatum. Striatal dopamine (DA) depletion in PD is considered to impede movement by inducing hypo- and hyperactivity in local spiny projection neurons (SPNs) of the direct (dSPNs) and indirect (iSPNs) pathways, respectively [1]. L-DOPA is proposed as the first-line medicine for treating PD patients. After decarboxylation, L-DOPA is converted to DA which acts on dopamine receptors within the striatum. Dopamine D1 receptors (D1R) and D2 receptors (D2R) are segregated in the dSPNs and iSPNs, respectively. The activation of D1R and D2R produces a synergistic stimulation of locomotion, explaining the strong anti-parkinsonian effect of L-DOPA.

DA and acetylcholine (ACh) exhibit numerous cooperative and reciprocal interactions within the striatum [2]. The striatum has the highest level of ACh in the brain, most of which originates from local cholinergic interneurons (ChIs) [3, 4]. DA can both activate and inhibit the activity of ChIs by acting on their D1/D5 receptors and D2R, respectively [5]. Interestingly, previous studies demonstrate that L-DOPA regulates the level of striatal acetylcholine (ACh), measured by the microdialysis method in parkinsonian mice. The induced alterations of ACh were controversial in parkinsonian conditions [6, 7], and its mechanism remains poorly understood. Cholinergic signals were considered to suppress dSPNs by activating expressed M4 muscarinic acetylcholine receptors (mAChRs) coupled Gi/o protein and activate iSPNs via post-synaptic M1 mAChRs coupled Gα protein. Human and rodent studies have reported striatal hypercholinergy following DA depletion, contributing to motor impairment [8–12]. Both optogenetic inhibition of ChIs and the application of mAChR antagonists significantly improved the motor deficits characterized by PD [13, 14]. Taken together, reducing cholinergic signaling in the striatum is traditionally considered to attenuate parkinsonian symptoms.

Interestingly, recent studies reported that striatal ACh triggered DA release via activating nicotinic acetylcholine receptors (nAChRs) expressed in distal axons of dopaminergic neurons through a synaptic-like axo-axonal transmission [15], and it could occur independently of somatic firing to provide distinct signaling [16]. Notably, there are a parallel decline in nAChRs expression with nigrostriatal damage [17], which has led to the long-standing belief that ACh-mediated DA release is negligible in advanced PD. It still remains poorly understood how ACh-induced DA release itself and to modulate striatal output in parkinsonian conditions. Dopaminergic and cholinergic neuromodulation in the striatum is essential for motor control, with prominent models proposing that DA and ACh release produce pro-kinetic and anti-kinetic effects, respectively. However, the results of fluorescence recordings indicated that both striatal DA and ACh systems were activated at spontaneous movement onsets with similar time courses [18]. Chemogenetic activation of ChIs was reported to increase locomotion function in intact rats [19]. Taken together, striatal ACh may produce pro-kinetic effects by triggering DA release in the parkinsonian condition.

Thus far, without neurotransmitter-level recordings that enable the in-vivo observation of interactions between DA and ACh, no definitive evidence reveals the comprehensive effects of interactions on striatal output and movement control in parkinsonian conditions. Here, we demonstrated the alterations of ACh levels following the systemic application of L-DOPA and related its mechanism. The striatal DA or ACh concentration in vivo was revealed by fiber photometry recordings with the genetically encoded fluorescent DA or ACh indicator [20, 21]. Compared with the microdialysis method, the fluorescent indicator can detect the distribution of neurotransmitters with a better spatiotemporal resolution [22]. To further confirm the effects of modulating striatal ACh on striatal output, we recorded in-vivo alterations of dSPNs and iSPNs activities and performed behavioral tests in parkinsonian mice.

## Methods

### Mice

Animal care and usage were conducted in strict accordance with institutional guidelines and government regulations. All experiments were approved by the Institutional Animal Care and Use Committee at ShanghaiTech University, China. Only male mice were used in this study. Male C57BL/6 mice, aged 7–8 weeks, were purchased from Shanghai Lingchang Biotech. Co., Ltd. (Shanghai, China). The BAC-transgenic D1-Cre [MMRRC Tg(Drd1a-Cre) EY262Gsat] and D2-Cre [MMRRC Tg(Drd2-Cre)ER44Gsat] mouse lines were obtained from MMRRC (Davis, California) [23]. The ChAT-ires-Cre mouse line was sourced from Jackson Laboratory (USA). Adult D1/D2/ChAT-Cre mice, aged 8–15 weeks, were used in this study. All mice were housed 4–5 per cage in a reversed 12-h light/dark cycle at 22° to 25 °C.

### Unilateral 6-OHDA model

The intraperitoneal injection(i.p.) with desipramine (25 mg/kg, Sigma-Aldrich) was performed 30 min before 6-OHDA infusion to prevent the lesioning of non-dopamine monoamine neurons by blocking serotonin and noradrenaline transporters. We anesthetized all mice with 1.5% isoflurane. The scalp was incised, and the fascia covering the skull was cleared using 3% hydrogen peroxide in saline. The bregma and lambda points were aligned to level the mouse's head. We stereotaxically inserted a needle into the SNc from Bregma (Anterior–Posterior(AP), − 3.1 mm; Medical-Lateral(ML), − 1.25 mm; and Dorsal–Ventral(DV), − 4.5 mm). The needle was connected to a syringe pump loaded with 6-OHDA (10ug/ul, Sigma-Aldrich), which was dissolved in sterile saline (0.9%) containing ascorbic acid (0.02%, Sigma-Aldrich). We infused 240 nl of 6-OHDA at a rate of 50–60 nl/min into SNc and keep the injection glass pipette in place for 10 min after the final infusion. After infusion, we administered 1 mL of lactated Ringer’s solution subcutaneously(s.c.) to prevent dehydration. Then, all mice were placed on a warm blanket to recover. In the first seven days, we monitored the body mass of each mouse and provided them with soft mouse chow and 5% glucose solution to aid recovery.

**Fig. 1:**
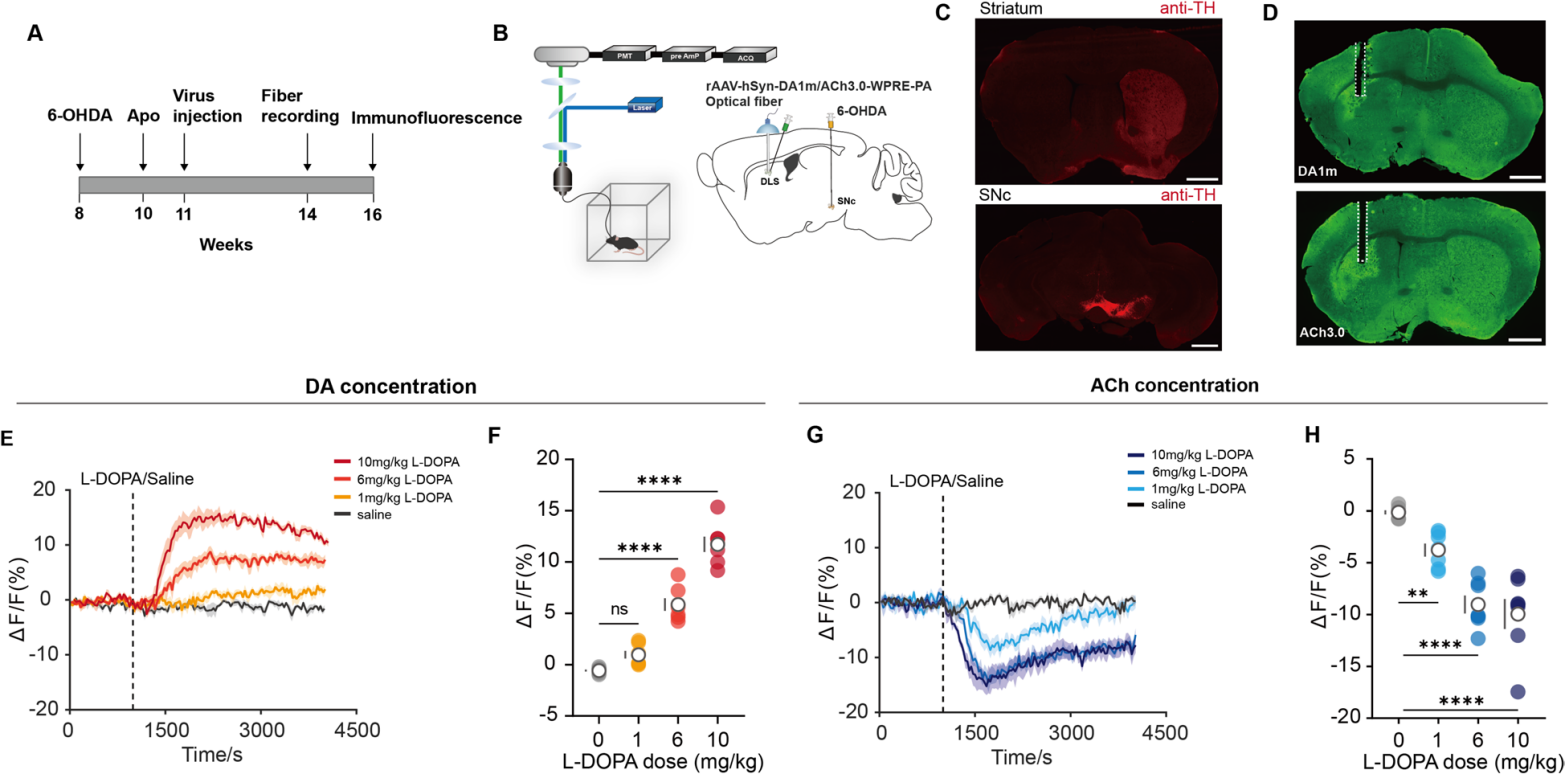
Effect of L-DOPA on DA and ACh concentration in the dorsolateral striatum of parkinsonian mice. **A** Schematic drawing of experimental timeline. **B** Schematic diagram of the fiber photometry recording system, 6-OHDA injection into SNc, viral strategy for DA1m or ACh3.0 expression, and optical fiber implantation into DLS. **C** Coronal striatal and midbrain section of TH immunoreactivity following unilateral 6-OHDA injection. Scale bar: 500um. **D** Image of DA1m/ ACh3.0 sensor and fiber channel in the coronal DLS section. Scale bar: 500um. **E** DA1m sensor signals from DLS aligned to the moment of the administration of L-DOPA and saline. Mean values are shown as a red-yellow line (L-DOPA) and a black line (saline), SEM, interval is shaded in red-yellow and gray. **F** Quantification of change in DA1m sensor signals after administration of saline and L-DOPA [n = 7, F(3,18) = 115.1, P < 0.0001, RM one-way ANOVA with Post hoc Bonferroni’s test]. **G** ACh3.0 sensor signals from DLS aligned to the moment of the administration of L-DOPA and saline. Mean values are shown as a blue line (L-DOPA) and a black line (saline), SEM, interval is shaded in blue and gray. **H** Quantification of change in ACh3.0 sensor signals after administration of saline and L-DOPA [n = 7, F(3,18) = 50.47, P < 0.0001, RM one-way ANOVA with Post hoc Bonferroni’s test]. Data are presented as the mean ± SEM. *, p < 0.05; **, p < 0.01; ***, p < 0.001; ****, p < 0.0001; ns, not significant; DA, dopamine; ACh, acetylcholine; DLS, dorsolateral striatum

All 6-OHDA-lesioned mice were evaluated in the apomorphine (Apo)-induced rotation test 2–3 weeks after surgery. Net contralateral turns were recorded during a 20-min period of rotational response (from 5 to 25 min) following the injection of apomorphine hydrochloride (0.05 mg/kg in 0.9% saline, s.c., Sigma-Aldrich) [24]. Raw data were collected by counting the number of rotations in the video. A rotation was defined as a complete 360° turn of the body without a change in head direction. Only animals exhibiting more than four turns/min were kept for the further experiment. Notably, striatal dopamine denervation and dopaminergic neurons in SNc were confirmed in each mouse through tyrosine hydroxylase(TH) immunohistochemistry. All mice included in the study exhibited an 80%-95% reduction in TH optical density in the dorsal striatum (Fig. 1C). All recording and behavioral experiments were conducted a minimum of 28 days after the lesion surgery.

### Surgery

Virus injections were performed using the same method as those for 6-OHDA infusion. rAAV-hSyn-DA1m-WPRE-PA [20], rAAV-hSyn-ACh3.0-WPRE-PA [21], rAAV-CAG-FLEX-jGCaMP7b-WPRE-SV40-PA, and rAAV-hSyn-DIO-hM3D(Gq)-mCherry-WPRE-hGH polyA were purchased from BrainVTA (Wuhan, China). Optical fiber and cannula were purchased from RWD Life Science Co., LTD (Shenzhen, China). 150 ~ 300 nl virus solution was injected into the ipsilateral dorsolateral striatum (DLS) (coordinate from Bregma: 0.3 mm AP, − 2.45 mm ML, − 3.2 mm DV) in the unilateral 6-OHDA-lesioned or sham-lesioned mice three weeks after the lesioned surgery. An optical fiber (200 um outside diameter, 0.37 numerical aperture) encased in a ceramic ferrule was gradually inserted into the DLS, with the tip positioned 0.2 mm above the viral injection sites. The fiber was then secured to the skull using dental cement.

For cannula implantation, the surgical method was the same as the previous statement in the lesioned surgery. The cannulas were implanted without solution injection and slowly inserted into the DLS (coordinate from Bregma: 0.3 mm AP, − 2.45 mm ML, − 2.7 mm DV).

For mice used in intra-striatal pharmacological infusions with simultaneous recording of DA1m or ACh3.0 signal in the DLS, 200 nl virus was injected at a depth of 3.23 mm form Bregma with optic fiber implant at AP 0.3 mm and ML − 2.9 mm at a − 8° angle and a depth of 3.03 mm. Then, a cannula was implanted at AP 0.3 mm and ML − 0.55 mm at a 30° angle and a depth of 3.31 mm ipsilateral to the implantation of the optic fiber; both were cemented to the skull using dental cement.

### Pharmacology

We dissolved the D_1_-like dopamine receptors (D1R)-selective agonist (SKF81297; 2.5 or 5 mg/kg; Sigma-Aldrich), D_2_-like dopamine receptors (D2R)-selective agonist (Quinpirole; 2.5 or 5 mg/kg; Sigma-Aldrich), D1R antagonist (SCH23390; 0.2 mg/kg; Sigma-Aldrich), D2R antagonist (Raclopride; 2 mg/kg; Sigma-Aldrich), L-DOPA (1, 6 or 10 mg/kg; Sigma-Aldrich) mixed with benserazide (15 mg/kg; Sigma-Aldrich), Donepezil (Den; 1 or 3 mg/kg; Selleck), Picrotoxin (PTX; 0.3 mg/mL; Tocris Bioscience), and Mecamylamine (Meca; 3 mg/kg and 50uM; MedChemExpress) in 0.9% saline solution. Except for Den (5ug/ul), Meca (50uM), and PTX (0.3 mg/mL) for intracerebral infusion, we administered other drugs to the mice systemically via intraperitoneal injection (10 ml/kg injection volume). The doses were chosen based on the published study [1, 25] and our evaluation of the dose-dependent effects of these drugs on striatal DA and ACh concentration in parkinsonian mice (Fig. 1E and G).

#### Fiber photometry recording

Fiber photometry recordings were performed in open-field chambers. Prior to the experiment, all mice were handled and habituated to the chamber and i.p. injections for three consecutive days. After a recovery period of 2–3 weeks, we recorded fluorescence signal of DA or ACh, as well as Ca^2+^ signals from dSPNs or iSPNs in DLS. The signal was acquired using a fiber photometry system, which included a 488-nm excitation laser, a 505- to 544-nm emission filter, and a photomultiplier tube (Fig. 1B). The laser power output was set to 10 to 30uW. Analog signals were digitalized at 100 Hz and recorded using a 1401 digitizer and Spike2 software (CED, Cambridge, UK).

For the fiber photometry experiments, the analysis method was previously described [26]. In this study, all data were analyzed using custom programs written in MATLAB (MathWorks, Version R2021a). Fluorescence change values (ΔF/F) were calculated as (F − F0)/F0, where F0 represented the baseline fluorescence signal averaged over a 1000-s control time window. The ΔF/F values were presented in average plots, with a shaded area representing the standard error of the mean (SEM). To obtain the statistical values, we extracted the average ΔF/F value from 3000–7000 s post-drug or saline injection as the treatment value. For the dual intervention group, the mean ΔF/F value from 2400–3000 s after the second injection was used as the treatment value.

### Open field test

All animals were placed in the center zone of a 38 × 38 × 38 cm open-field chamber, equipped with dim lighting and a fan, for 40 min. Spontaneous locomotion was recorded using a video tracking system and analyzed with the JL-Behv-LAG-4 video analysis software (Jiliang Software Technology Co., Ltd., Shanghai, China). The total distance traveled within the chamber was tracked and subsequently analyzed. Data were collected in 10-min intervals.

### Rotations

#### Net ipsilateral rotations

Mice with severe lesions, characterized by an extensive depletion exceeding 80% of TH^+^ innervation within the striatum, exhibited significant spontaneous rotation towards the lesioned side [27, 28]. Full-body turns were quantified, and rotation toward the lesioned side was assigned a negative value. Data was collected in 10-min bins. We manually scored the number of ipsilateral rotations based on a video. The video materials had undergone a renaming process with the intent of obfuscating the initial grouping from data analysts.

All mice underwent habituation to the open-field chamber and intracutaneous injections for three consecutive days prior to the experiment. After the habituation period, there were two consecutive days of testing, during which mice received injections of Den (2.5ug, 5ug/ul, i.c.) or saline (Fig. S5B).

##### Turing *bias* assay

DA replacement therapies can induce dose-dependent turning behavior, which serves as the standard measure of therapeutic efficacy in parkinsonian mice [1, 29]. Full-body turns were quantified, and rotation toward the contralateral side of the lesion was assigned a positive value. We manually recorded the number of rotations made by mice, both ipsilateral and contralateral to the left cerebral hemisphere, during a 40-min recording session in the open-field chamber. Video materials were renamed to obscure the original grouping from data analysts. The rotational bias (contralateral minus ipsilateral turns) for each mouse was calculated by subtracting the total number of rotations in each direction. The rational bias of each mouse was recorded in 10- minute intervals.

All mice were habituated to the chamber and receive i.p./i.c. injections for three consecutive days before the actual experiment. Following the habituation period, there were three consecutive days of testing (Fig. 5B), during which mice received intraperitoneal injections of saline combined with the intracerebral infusion of saline on the first day. On the second or third day, animals received L-DOPA injection (6 mg/kg, i.p.) combined with the intracerebral infusion of saline (500 nl) or Den (2.5ug, 500 nl) according to a randomized crossover design (Fig. 5B), respectively.

#### Dyskinesia assay

The abnormal involuntary movement score (AIMs) is a widely recognized metric for assessing dyskinesia levels in parkinsonian mice subjected to various drug treatments [30]. Briefly, following systemic injection of L-DOPA, we assessed each mouse’s AIMs every 20 min over a 3-h period by observing its behavior for 60 s. We specifically monitored the occurrence of abnormal movements in (i)the axial region, (ii) the limbs, and (iii) the orofacial area. Within each 60-s interval, we assigned a score ranging from 0 to 4 for each category (i–iii). A score of '0' indicated no abnormalities, while scores of '1' or '2' indicated abnormal behaviors lasting less than 30 s or more than 30 s, respectively. A score of '3' indicated abnormal behavior lasting the entire 60 s but interruptible by an external stimulus (e.g., a loud clap), and '4' indicated uninterrupted abnormal behavior for the full 60 s. The total AIMs value for each 60-s interval was obtained by summing the individual category scores.

#### Histology and immunohistochemistry

All mice were anesthetized with tribromoethanol (250 mg/kg, intraperitoneally) and then perfused through the heart with 0.9% saline. After the blood was drained, all mice were fixed with 4% paraformaldehyde (PFA). Following decapitation, the head was immersed in 4% PFA at room temperature overnight. The following day, the brains were harvested and immersed in 30% sucrose in 0.1 M phosphate-buffered saline (PBS, pH 7.4) at 4 °C for 24 to 48 h. Coronal brain Sects. (30 um) containing the striatum and SNc were sliced using a cryostat (Leica CM3050s). The sections were washed with 0.1 M PBS, blocked in a solution containing 0.3% Triton X-100 and 5% bovine serum albumin in 0.1 M PBS for 2–4 h at 4℃, and then incubated with the primary antibody (rabbit anti-TH, 1:1000; Invitrogen) in the blocking buffer at 4℃ for 24 to 48 h. After three washes with 0.1 M PBS, the sections were incubated with donkey anti-rabbit IgG H&L secondary antibody conjugated to Alexa Fluor-594 (1:1000; Jackson ImmunoResearch) for 2–4 h at room temperature. The nucleus was stained with 4′,6-diamidine-2-phenindoles (DAPI), and the sections were mounted in glycerin and covered with coverslips, ensuring they were sealed in place.

Fluorescent images were obtained with an Olympus VS120 microscope. The data of fluorescent signals were extracted by Image J (Version 1.53q) and Qupath software (Version 0.3.0) [31].

### Statistical analysis

Data were analyzed with GraphPad Prism software (Version 9.0; GraphPad Software, San Diego, CA, USA) using ANOVA, repeated measures (RM), and post hoc Bonferroni’s test, corrected for multiple comparisons. For between-group comparisons, non-parametric tests (i.e., the Wilcoxon matched-pairs signed rank test) was used. Correlation analyses were conducted using the Spearman's correlation coefficient analysis. All data are expressed as means ± SEM. For all analyses, *P* < 0.05 was considered the level of statistical significance.

## Results

### L-DOPA increased DA and inhibited ACh release of the dorsolateral striatum only in parkinsonian condition

To monitor the temporal changes of DA following the systemic application of L-DOPA in the parkinsonian and healthy condition (Fig. 1A, C), we infused rAAV-hSyn-DA1m-WPRE-PA(DA1m) virus to DLS in the 6-OHDA-lesioned or sham-lesioned mice (Fig. 1B and Fig.S1A). We next used fiber photometry to record fluorescence signals revealing DA concentration in free-moving mice. We found that the intraperitoneal injection of medium- and high-dose L-DOPA (6 and 10 mg/kg, i.p.) significantly increased DA in parkinsonian mice, presenting dose-dependent to modulate striatal DA production (Fig. 1E, F). Upon observing that DA production in the striatum was correspondingly enhanced as the dose of L-DOPA increased in parkinsonian mice, we investigated the effects of high-dose L-DOPA on DLS DA levels in healthy mice. In sham-lesioned mice, the high-dose L-DOPA (10 mg/kg, i.p.) failed to enhance DA concentration (Fig.S1B and S1C). To assess the alteration in ACh levels in parkinsonian and healthy state, we infused rAAV-hSyn-ACh3.0-WPRE-PA (ACh3.0) virus into DLS in the 6-OHDA-lesioned or sham-lesioned mice (Fig. 1B and Fig.S1D). By using fiber photometry recordings, we found dose-dependent L-DOPA significantly inhibited ACh release in DLS compared with saline (Fig. 1G, H). Upon observing that higher doses of L-DOPA resulted in less ACh release in the striatum of parkinsonian mice, we investigated the effects of high-dose L-DOPA on DLS ACh levels in healthy mice. The high-dose L-DOPA (10 mg/kg, i.p.) failed to reduce ACh concentration in the sham-lesioned mice (Fig.S1E and S1F). Taken together, L-DOPA only enhanced the DA level of the dopamine-depleted striatum and performed an inhibitory modulation on ACh release.

### DA inhibits DLS ACh release through D2R and D1R in parkinsonian mice

**Fig. 2:**
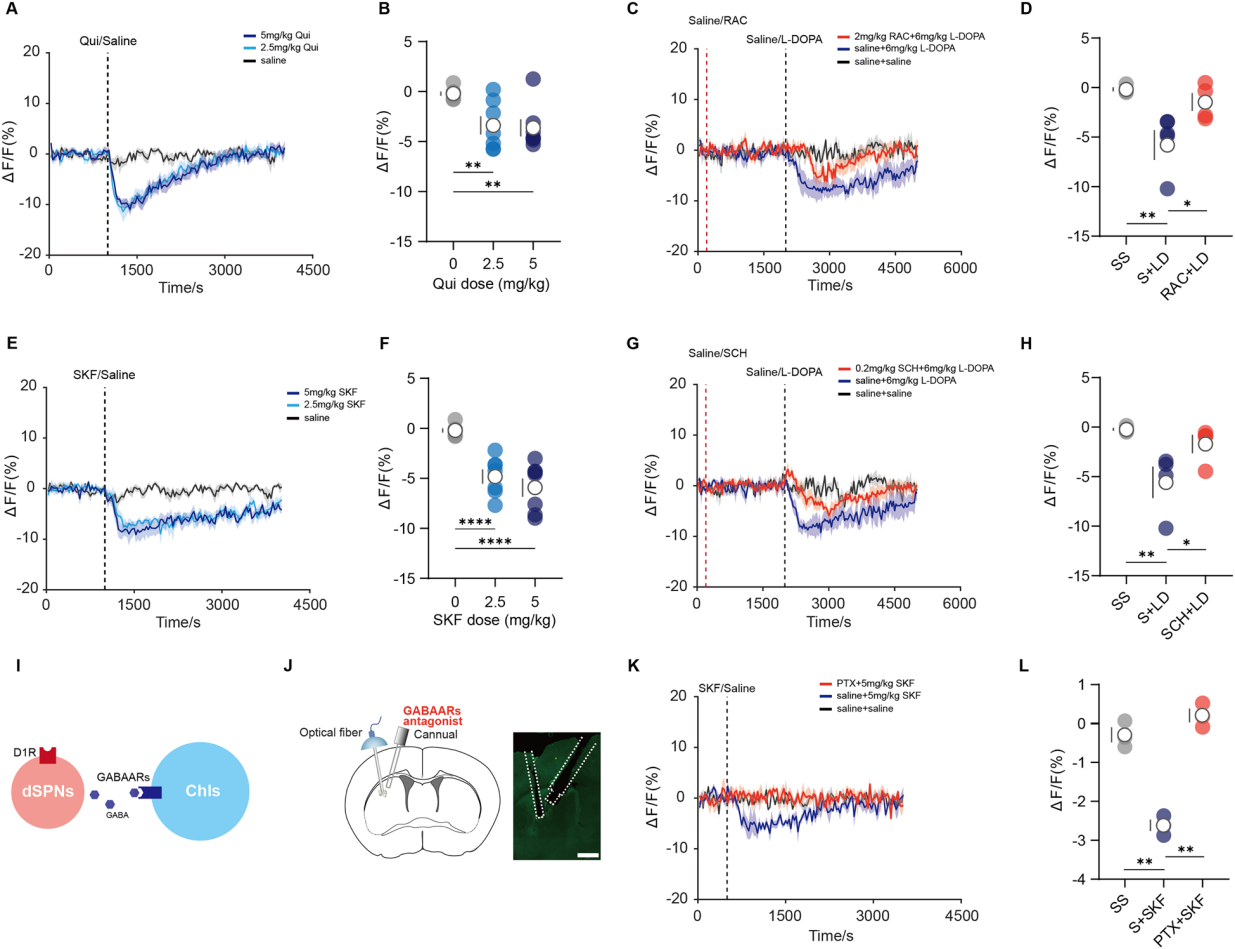
Activation of D1R and D2R regulated ACh dynamics in the dorsolateral striatum. **A** and **C** ACh3.0 sensor signals from DLS of 6-OHDA-lesioned mice aligned to the moment of the administration of D2R-selective agonist (Quinpirole, Qui), D2R-selective antagonist (Raclopride, RAC), L-DOPA, and saline. Mean values are shown as a blue line (Qui and saline-L-DOPA), a red line (RAC-L-DOPA), and a black line (saline), SEM, interval is shaded in red, blue, and gray. **B** Quantification of change in ACh3.0 sensor signals after administration of saline and Qui [n = 7, F(2,12) = 9.474, P = 0.0034, RM one-way ANOVA with Post hoc Bonferroni’s test]. **D** Quantification of the change in ACh3.0 sensor signals after the second administration of saline and L-DOPA [n = 4, F(2,6) = 12.32, P = 0.0075, RM one-way ANOVA with Post hoc Bonferroni’s test]. **E** and **G** ACh3.0 sensor signals from DLS of 6-OHDA-lesioned mice aligned to the moment of the administration of D1R-selective agonist (SKF-81297, SKF), D1R-selective antagonist (SCH23390, SCH), L-DOPA, and saline. Mean values are shown as a blue line (SKF and saline-L-DOPA), a red line (SCH-L-DOPA), and a black line (saline), SEM, interval is shaded in red, blue, and gray. **F** Quantification of change in ACh3.0 sensor signals after administration of saline and SKF [n = 7, F(2,12) = 33.98, P < 0.0001, RM one-way ANOVA with Post hoc Bonferroni’s test]. **H** Quantification of the change in ACh3.0 sensor signals after the second administration of saline and L-DOPA [n = 4, F(2,6) = 11.47, P = 0.0089, RM one-way ANOVA with Post hoc Bonferroni’s test]. **I** Diagram illustrating activation of D1R promoting GABA release from dSPNs acting on GABAARs on ChIs. **J** Left, schematic diagram of the fiber photometry recording system and local drugs infusion. Right, Image of ACh3.0 sensor, fiber channel, and cannula channel in the coronal DLS section. Scale bar: 500um. **K** ACh3.0 sensor signals from DLS aligned to the moment of the systemic administration of SKF and saline. Mean values are shown as a red line (PTX-SKF) a blue line (saline-SKF) and a black line (saline-saline), SEM, interval is shaded in red, blue, and gray. **L** Quantification of the change in ACh3.0 sensor signals after the second administration of saline and SKF [n = 3, F(2,4) = 59.58, P = 0.0011, RM one-way ANOVA with Post hoc Bonferroni’s test]

Enhanced DA may indirectly or directly modulate striatal ACh levels through the activation of D1R and D2R located on several microcircuit elements [5]. We sought to assess how selective activation of D2R or D1R compared to combined activation with L-DOPA. Previous studies reported that striatal dopaminergic signaling could inhibit ChIs activity via expressed D2R [5]. As expected, the systemic application of the D2R agonist (Quinpirole, Qui) resulted in the inhibitory regulation of striatal ACh release in parkinsonian mice (Fig. 2A, B). This effect was comparable to the findings observed in the sham-lesioned mice (Fig.S2A and S2B). Pretreatment with the D2R antagonist (Raclopride, RAC) for 30 min effectively decreased the power of L-DOPA-induced ACh release events in parkinsonian mice (Fig. 2C, D). Remarkably, we found that the D1R agonist (SKF81297, SKF, i.p.) decreased striatal ACh levels in parkinsonian mice but not in sham-lesioned mice (Fig. 2E, F, and Fig.S2C). The pretreatment of D1R antagonist (SCH23390, SCH, i.p.) effectively decreased the power of L-DOPA-induced ACh release events in parkinsonian mice (Fig. 2G, H).

### GABAARs were required for the activation of D1R-induced inhibition of ACh signals

The burst firing in ChIs is triggered by DA via expressed D1/D5R in intact mice [5]. More than half of the synapses on ChIs arise from local GABAergic neurons, including SPNs and GABAergic interneurons [32–34]. Notably, L-DOPA and D1R agonists are potent activators of dSPNs [1]. Therefore, D1R-mediated activation of dSPNs may lead to the release of γ-aminobutyric acid (GABA), which acted on GABAARs of ChIs, subsequently downregulating their activity (Fig. 2I). To determine whether the activation of D1R signaling on ACh release was regulated by striatal GABAARs in vivo, we infused PTX (0.3 mg/mL, 500 nl), a GABAARs antagonist, into the DLS before the systemic application of SKF while locally imaging ACh in vivo using fiber photometry (Fig. 2J). As expected, this manipulation effectively blocked the detection of SKF-evoked ACh release events in parkinsonian mice (Fig. 2K, L).

### Restoring ACh levels downregulated by L-DOPA triggered more DA release through nAChRs

**Fig. 3:**
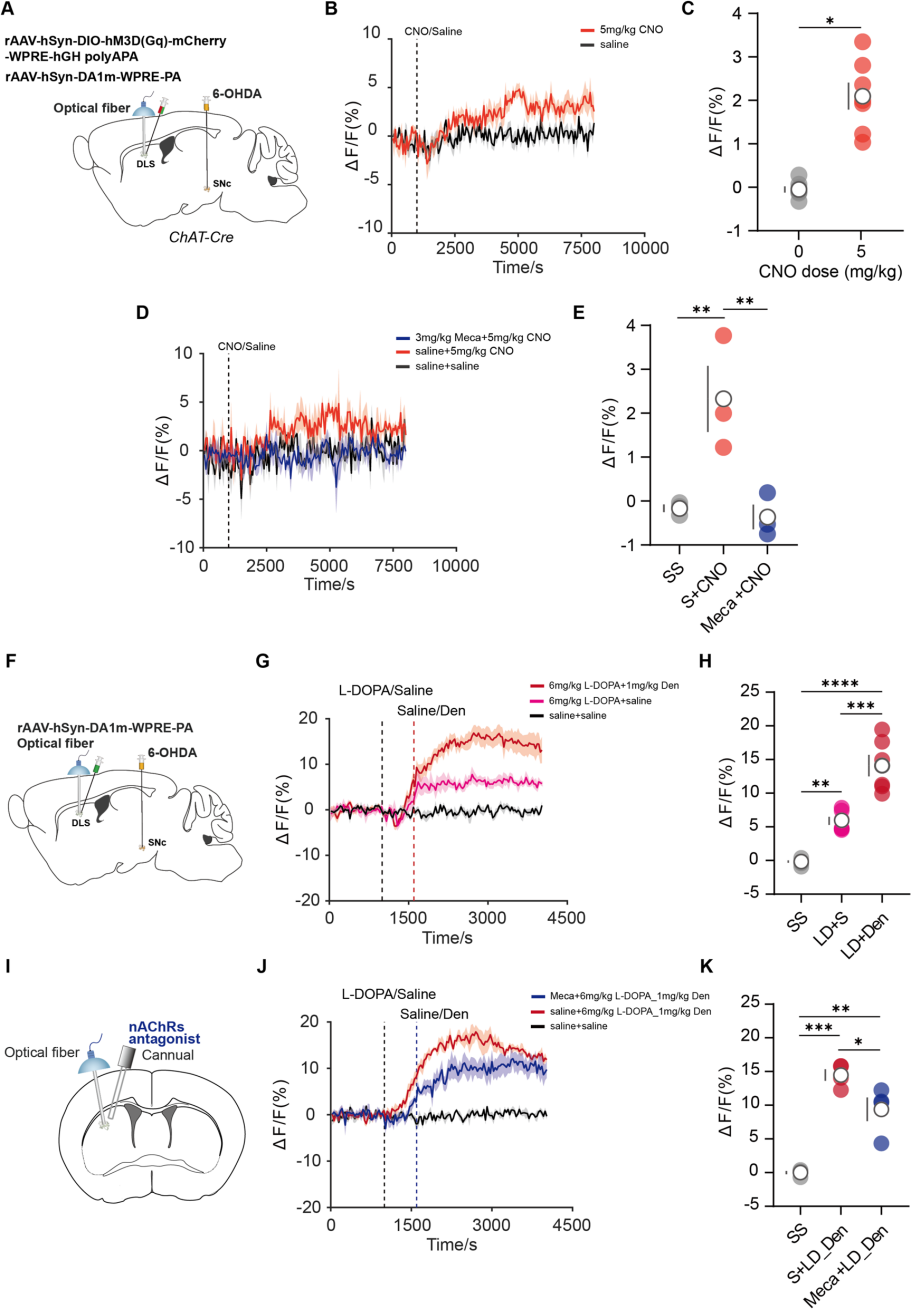
Restoration of striatal ACh level triggered more DA release. **A** Schematic diagram of the fiber photometry recording system, 6-OHDA injection into SNc, viral strategy for DIO-hM3D(Gq) and DA1m expression, and optical fiber implantation into DLS. **B** and **D** DA1m sensor signals from DLS aligned to the moment of the administration of Meca, CNO and saline. Mean values are shown as a blue line (Meca-CNO), a red line (CNO), and a black line (saline). SEM, interval is shaded in red and gray. **C** Quantification of change in DA1m sensor signals after administration of saline and CNO [n = 7, Wilcoxon matched-pairs signed rank test, P = 0.0156]. **E** Quantification of change in DA1m sensor signals after administration of saline and CNO [n = 3, F(2,4) = 16.18, P = 0.0121, RM one-way ANOVA with Bonferroni’s post-hoc test]. **F** Schematic diagram of the fiber photometry recording system, 6-OHDA injection into SNc, viral strategy for DA1m or ACh3.0 expression, and optical fiber implantation into DLS. **G** and **J** DA1m sensor signals from DLS aligned to the moment of the administration of Meca, L-DOPA, Den, and saline. Mean values are shown as a blue line (Meca-L-DOPA-Den), a red line (L-DOPA-Den), a pink line (L-DOPA-saline), and a black line (saline-saline), SEM, interval is shaded in red, pink, blue, and gray. **H** Quantification of change in DA1m sensor signals after administration of saline and Den [n = 6, F(2,10) = 57.6, P < 0.0001, RM one-way ANOVA with Bonferroni’s post-hoc test]. **I** Schematic diagram of the fiber photometry recording system and local drugs infusion. **K** Quantification of change in DA1m sensor signals after administration of saline and Den [n = 4, F(2,6) = 45.32, P = 0.0002, RM one-way ANOVA with Bonferroni’s post-hoc test]

To investigate whether enhancing striatal ACh triggered DA release by modulating nAChRs expressed in the remaining DA axons, we infused Cre-dependent hM3D(Gq) receptor and DA1m virus into the DLS of 6-OHDA-lesioned ChAT-Cre mice. This led to hM3Dq–mediated activation of ChIs while simultaneously imaging DA1m signals in the ipsilateral DLS using photometry (Fig. 3A). The activation of ChIs by 5 mg/kg CNO significantly increase DA concentration (Fig. 3B, C), which was blocked with the systemic pre-treatment of 3 mg/kg Meca (Fig. 3D, E). Next, we tested whether increasing striatal ACh levels through pharmacological intervention inhibiting acetylcholinesterase (AChE) activity modulated DA production. To monitor the temporal changes of DLS ACh following the systemic administration of Den, an AChE inhibitor, in the parkinsonian condition, we infused ACh3.0 virus to the DLS in the 6-OHDA-lesioned mice (Fig.S3A). We found that both low- and high-dose Den (1 mg/kg and 3 mg/kg) increased DLS ACh (Fig.S3B and S3C). Next, we infused the DA1m virus into the DLS in the 6-OHDA-lesioned mice to investigate the effect of increasing striatal ACh on local DA level (Fig.S3D). We observed that high-dose Den significantly increased DLS DA compared with saline; applying low-dose Den failed to trigger DA release, as revealed by fluorescent signals (Fig.S3E and S3F).

Next, we tested whether restoring striatal ACh level, downregulated by L-DOPA, enhanced the effect of L-DOPA. Based on our results, ACh reached its peak level approximately 10 min after L-DOPA administration. Thus, we applied Den 10 min after the injection of 6 mg/kg L-DOPA (Fig.S4A and S4B). To elucidate the role of ACh levels downregulated by L-DOPA, the optimal intervention strategy involved restoring ACh levels to baseline following L-DOPA administration. We found that a low-dose Den (i.p.) was sufficient to restore ACh level downregulated by L-DOPA (Fig.S4C and S4D). Interestingly, low-dose Den significantly triggered more DA release, as revealed by fluorescent signals (Fig. 3F–H). The intra-striatal infusion of Meca (50uM, 500 nl) effectively decreased the power of Den-induced more DA release events in parkinsonian mice (Fig. 3I–K).

### Restoring ACh levels bidirectionally regulated the activity of SPNs induced by L-DOPA in parkinsonian mice

**Fig. 4:**
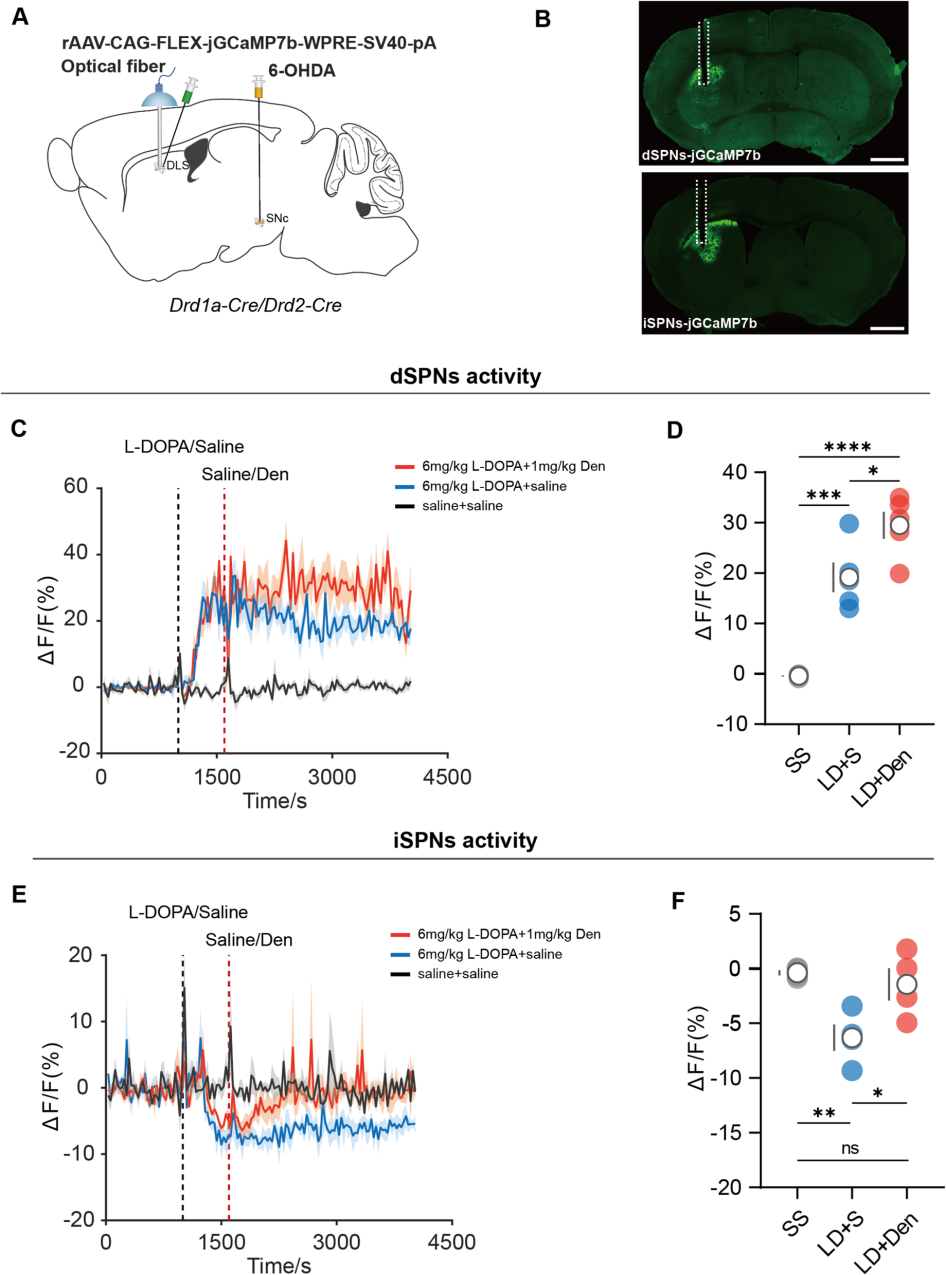
Enhancing striatal ACh modulated SPNs activity evoked by L-DOPA. **A** Schematic diagram of the fiber photometry recording, 6-OHDA injection into SNc, viral strategy for jGCaMP7b expression, and optical fiber implantation into DLS. **B** Image of dSPNs/iSPNs and fiber channel in the coronal DLS section. Scale bar: 500um. **C** jGCaMP7b signals from DLS dSPNs aligned to the moment of the administration of L-DOPA, Den, and saline. Mean values are shown as a red line (L-DOPA-Den), a blue line (L-DOPA-saline), and a black line (saline-saline), SEM, interval is shaded in red, blue, and gray. **D** Quantification of change in jGCaMP7b signals after administration of saline and Den [n = 5, F (2,8) = 49.37, P < 0.0001, RM one-way ANOVA with post-hoc Bonferroni’s test]. **E** jGCaMP7b signals from DLS iSPNs aligned to the moment of the administration of L-DOPA, Den, and saline. Mean values are shown as a red line (L-DOPA-Den), a blue line (L-DOPA-saline), and a black line (saline-saline), SEM, interval is shaded in red, blue, and gray. **F** Quantification of change in jGCaMP7b signals after administration of saline and Den [n = 4, F (2,6) = 12.73, P = 0.0069, RM one-way ANOVA with post-hoc Bonferroni’s test]. SPNs, spiny projection neurons; dSPNs, spiny projection neurons in the direct pathways; iSPNs, spiny projection neurons in the indirect pathways

Calcium imaging technique have been a flexible approach for characterizing a range of neuronal events in vivo [35, 36]. To visualize the dSPNs and iSPNs activity, we infused rAAV-CAG-FLEX-jGCaMP7b-WPRE-SV40-PA virus into the DLS of 6-OHDA-lesioned D1-Cre and D2-Cre mice, respectively (Fig. 4A, B). Consistent with the previous results [1, 37], we found L-DOPA increased dSPNs activity and decreased iSPNs activity (Fig. 4C, E). We applied Den (1 mg/kg, i.p.) 10 min after the systemic injection of 6 mg/kg L-DOPA. Enhanced dSPNs activity was observed after applying Den (Fig. 4C, D). In contrast, Den increased the activity of iSPNs, blunting the suppression induced by L-DOPA (Fig. 4E, F).

### Increasing striatal ACh facilitated rotational responses toward L-DOPA

Contralateral rotations in unilaterally 6-OHDA-lesioned mice serve as a standard measure for evaluating the anti-parkinsonian potential of compounds [1, 29, 38–40]. To investigate the modulation of ACh-mediated additional DA release on motor deficits in PD, we conducted a typical behavioral experiment called the turning bias assay. We delivered Den or saline into the DLS via an implanted cannula (Fig. S5A, S5B, and S5C). Local infusion of Den did not induce contralateral rotations but reduced ipsilateral net rotations in parkinsonian mice (Fig.S5D and S5E). Moreover, it did not significantly affect traveled distance (Fig.S5F and S5G).

**Fig. 5:**
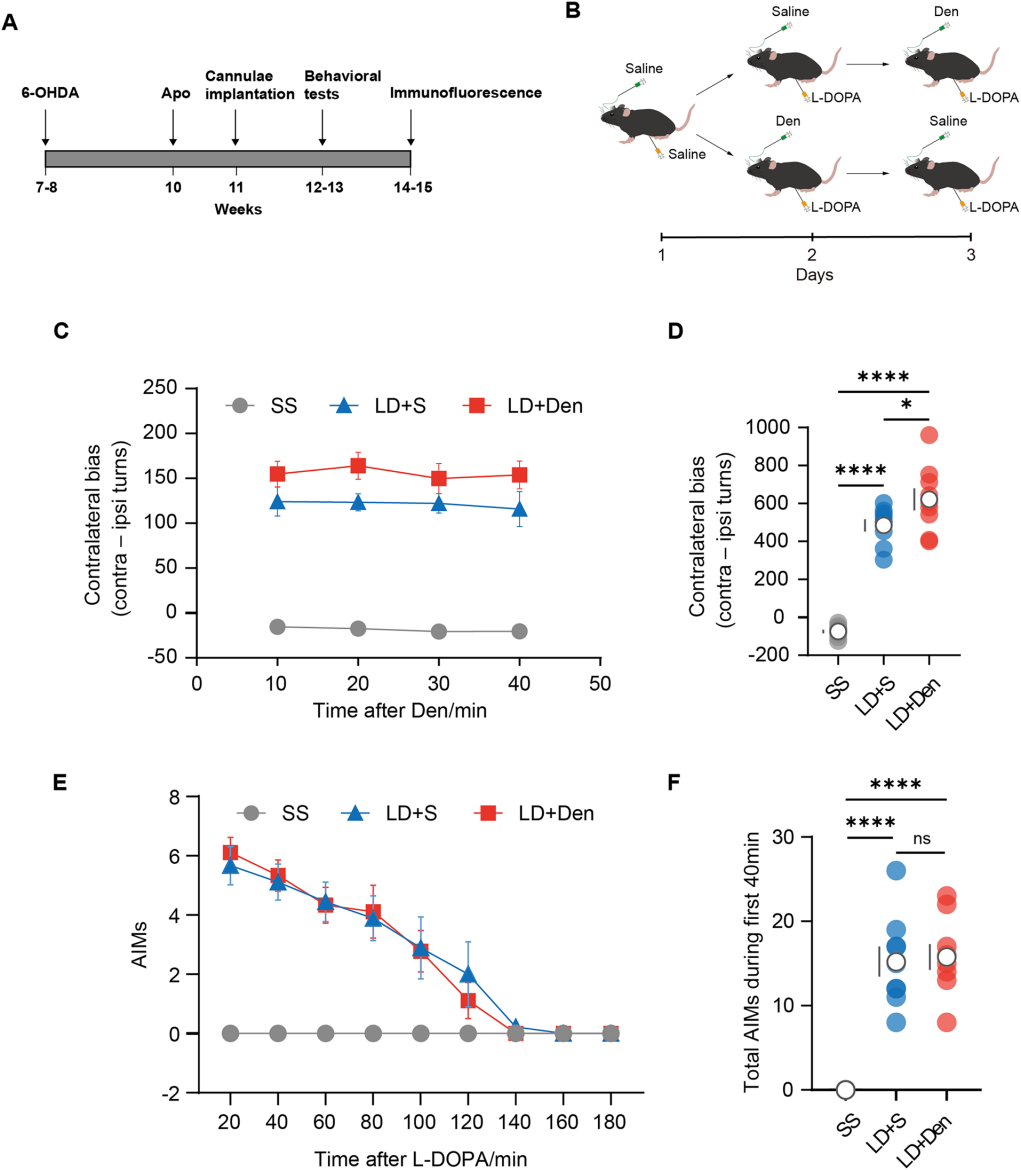
Specific upregulation of DLS ACh enhanced the effects of L-DOPA in parkinsonian mice. **A** Schematic drawing of experimental timeline. **B** Schematic diagram of the strategy of drugs injection for behavioral test (rotational behavior and dyskinesia). **C** Time trace showing effects of the different strategies of drugs injection on the contralateral rotational bias of mice, scored in 10-min time bins. **D** Comparison of the rotational bias in the first 40 min after administration of the different drugs combination [n = 9, F (2,16) = 149.0, P < 0.0001, RM one-way ANOVA with Bonferroni’s post-hoc test]. **E** Time trace showing effects of the different strategies of drugs injection on total AIMS values, scored in 20-min time bins. **F** Comparison of the total AIMs values in the first 60 min after administration of the different drugs combination [n = 9, F (2,16) = 72.85, P < 0.0001, RM one-way ANOVA with Bonferroni’s post-hoc test].

To restore striatal ACh levels following L-DOPA administration in parkinsonian mice, either Den or saline was intracerebrally injected (i.c.) 10 min after the systemic administration of L-DOPA (Fig. 5A, B). We observed that Den (2.5ug, 5ug/ul, i.c.) induced more contralateral rotations, augmenting the effects of L-DOPA (Fig. 5C, D). Additionally, it failed to exacerbate dyskinesia induced by L-DOPA (Fig. 5E, F).

## Discussion

Using fiber recording with genetically encoded fluorescent DA or ACh indicator, we observed that L-DOPA increased striatal DA and reduced local ACh in parkinsonian conditions. L-DOPA activated D1R- and D2R-related signaling, inducing alterations in ACh dynamics in parkinsonian mice. The activation of GABAARs contributed to D1R agonists evoked ACh event in parkinsonian mice. Restoring the ACh level modulated by L-DOPA triggered more DA release, which was blocked by intra-striatal infusion of nAChRs antagonist. Increasing striatal ACh enhanced dSPNs activity and blunted iSPN activity evoked by L-DOPA. Behavioral tests confirmed that enhancing DLS ACh facilities response to L-DOPA in parkinsonian mice. Our research proposes that DA-mediated inhibition of ACh release diminishes the response to L-DOPA in parkinsonian mice. Augmenting striatal ACh levels may enhance L-DOPA utilization in the brains of PD patients through modulating striatal DA tone. Additionally, our results suggest the striatal ACh might play a pro-kinetic role in motor regulation, highly dependent on DA tone.

Current pharmacological treatments for PD primarily aim to ameliorate symptoms by replenishing dopamine (DA) levels within the striatum [41]. The combination of Carbidopa with Levodopa (L-DOPA) serves to inhibit the peripheral metabolism of L-DOPA, thereby enhancing its bioavailability in the brain. Catechol-O-methyltransferase (COMT) inhibitors function by inhibiting the enzyme COMT, which degrades L-DOPA in peripheral tissues, thus extending the duration of L-DOPA's therapeutic effects. Monoamine oxidase B (MAO-B) inhibitors prevent the enzymatic breakdown of striatal DA, leading to increased DA levels and prolonged dopaminergic activity. Amantadine exerts its therapeutic effects through multiple mechanisms, including the augmentation of DA release, inhibition of DA reuptake, and antagonism of NMDA receptors, collectively contributing to symptom alleviation in PD. DA release can be modulated by striatal ACh signals. In the advanced stages of PD, the activation of nAChRs is proposed to has a markedly limited impact on DA signaling [2]. Interestingly, our study revealed that restoring ACh levels following L-DOPA treatment can enhance its effect by promoting striatal DA release in vivo, a process mediated by nAChRs. Our findings indicate that the cholinergic system within the striatum may present a promising target for augmenting the therapeutic efficacy of L-DOPA in PD patients.

Consistent with the findings of Parker et al. study [1], L-DOPA induced contralateral rotations in the mice model of our study, impeding behavioral assessments in open field, rotarod, and forelimb movement tests. Contralateral rotations in unilaterally 6-OHDA–lesioned mice serve as a customary metric for evaluating the anti-parkinsonian potential of compounds. [29, 38–40] Drugs-induced contralateral rotation is considered to directly stimulate postsynaptic DA receptors [39]; L-DOPA induces contralateral turning by producing extracellular DA to stimulate postsynaptic dopamine receptors. Previous studies confirmed D1R agonists, D2R agonists and L-DOPA induce contralateral rotations by restoring abnormal SPNs activity in parkinsonian mice [1]. Opponents tend to consider contralateral rotation as indicative of dyskinesia. Contralateral rotation can be triggered at very high levels by antiparkinsonian drugs with low dyskinesiogenic potential, like bromocriptine [42]. Amantadine, an anti-dyskinesia drug, markedly attenuated AIMs but do not influence rotational behavior [42]. Additionally, our study demonstrated that local infusion of Den in the DLS failed to worsen dyskinesia induced by acute application of L-DOPA.

In our study, changes in SPN activity were evaluated using calcium imaging technique [35, 36], a flexible approach for characterizing various neuronal events in vivo. Consistent with previous studies [1, 37], our results reveled that L-DOPA bidirectionally modulated dSPNs and iSPNs activity; restoring striatal ACh level following L-DOPA treatment resulted in the enhancement of SPNs activity. Notably, Optogenetic and chemogenetic studies support the notion that activated dSPNs improve parkinsonian motor deficits, while activation of iSPNs exacerbates bradykinesia [43, 44]. The behavioral tests confirmed that Den-induced changes contributed to a further improvement in motor deficits of PD following the administration of L-DOPA, which indicated that enhanced cholinergic signaling facilitated the effects of L-DOPA by further activating dSPNs.

The activity of iSPNs can be modulated by both striatal ACh and DA [2]. Our study found that the administration of Den blunted the suppression of iSPN activity induced by L-DOPA. Cholinergic signals activate iSPNs by stimulating their expressed M1 mAChR. Tozzi et al. study has reported that D2R stimulation produced a reduction of excitatory postsynaptic response (EPSC) in striatal iSPNs of DA-depleted mice, while the presence of an M1 mAChR antagonist prevents the effect of D2R agonists [45]. The pretreatment with the D2R antagonist (sulpiride) failed to decrease contralateral rotations induced by the intra-striatal injection of pirenzepine in WT mice [46]. These pieces of evidence suggest that M1 mAChR may have a critical role in mediating the D2R-dependent reduction of the EPSC amplitude in iSPNs.

The dopaminergic neurons in the SNc received cholinergic projection originating in the mesopontine nuclei and are activated via expressed nAChRs. [47, 48] Chemogenetic activation of cholinergic neurons in the pedunculopontine nucleus can evoke striatal DA release, as assessed by PET imaging in parkinsonian rats [49].Recent studies demonstrate that cholinergic signaling onto nAChRs expressed in dopamine axons triggers DA release, and this progress occurs independently of somatic firing [16]. Taken together, ACh-induced striatal DA release can occur either by activating dopaminergic neurons in the SNc or by modulating local DA exons. Our study had demonstrated that enhancing striatal ACh could trigger local DA release by modulating nAChRs expressed in the remaining dopaminergic axons in parkinsonian mice. Interestingly, a low dose of Den failed to trigger striatal DA release, but it could stimulate more DA release with L-DOPA supplementation, and the Den-evoked effect was decease by local infusion of Meca. L-DOPA is taken up by dopaminergic terminals, converted to DA, sequestered within vesicles, and subsequently released in an activity-dependent fashion. Electrochemical cytometry reveals the quantification of DA molecules within individual synaptic vesicles directly sampled from brain tissue, with vesicular content showing a significant time-dependent increase following L-DOPA administration [50, 51]. We reasonably speculate that there is an increase in DA in the residual terminals with L-DOPA supplementation, allowing the effects of ACh-mediated DA release to be detected. The parkinsonian mice had at least 4% dopaminergic neurons remaining in the lesioned SNc relative to the contralateral hemisphere. A high dose of Den may increase striatal DA by activating dopaminergic neurons and striatal dopamine axon in parkinsonian mice.

L-DOPA is the mainstay therapy for treating motor deficits of PD. AChE inhibitors are the first-line medicine in PD patients with cognitive deficits [52]. The study raised by Aarsland et al. report that the systemic administration of Den do not modulate response to L-DOPA in PD patients [52]. This observation may be related to its regulation of other pathways within the basal ganglia circuit; previous study demonstrates that cholinergic projections from the brainstem activate mAChR in the pre-synapse of SNr, which attenuates the effects of the D1R agonists [53]. Our results indicated that Den amplified the effects of L-DOPA at the striatal level. The systemic administration of Den simultaneously modulates cholinergic signaling in the striatum and SNr; cholinergic transmission in the SNr may attenuate the effect of striatal output signals.

The clinically therapeutic drugs and disease states should be tested to standardize their methodology. Techniques for neuronal population analysis are being developed to quantify, monitor, and decode these computations. Due to the development of in vivo tools for tracking neurotransmitters, their dynamics in disease can be assessed after applying therapeutic drugs. In vivo observation will provide valuable information regarding the complex process underlying intercellular communication and the induced changes in neural activity. Meanwhile, recordings in vitro would help to confirm the specific mechanisms of the pathological changes from animal models and the actions of clinically useful drugs.

## Conclusions

In summary, our study indicated that L-DOPA increased striatal DA and inhibited ACh release only in parkinsonian conditions. Interestingly, restoring the ACh level further enhanced striatal DA release evoked by L-DOPA. The results of behavioral tests further confirmed that enhancing striatal ACh facilitated the effects of L-DOPA in experimental parkinsonism by increasing dSPNs activity. Our results suggest that enhancing striatal ACh can exert pro-kinetic effects in parkinsonian mice, which may be highly dependent on striatal DA tone. Our study supported that the striatal ACh is not purely exerting anti-kinetic effects in parkinsonian mice. The ACh system may be a striatal regulator to regulate SPNs activity by exerting anti-kinetic effects via activating cholinergic pathways or pro-kinetic via triggering striatal DA release. The distinctive role of the coordination between striatal DA and ACh in regulating motor production was further emphasized by our results. Our findings provided a base of evidence for the development of new strategies to enhance striatal DA in patients with PD.

## Supplementary Information

Supplementary material 1: Supplementary material includes Fig S1, S2, S3, S4, and S5.

## Data Availability

All data of this study are available from the corresponding authors on reasonable request. The original MATLAB code used in this paper is shown in the Zhu et al. study [54].
