# Supplementary material for: Enhancing striatal acetylcholine facilitates dopamine release and striatal output in parkinsonian mice
Source: Cell Biosci. 2024 Dec 3;14:146. doi: 10.1186/s13578-024-01328-z (PMC11616140; doi:10.1186/s13578-024-01328-z)
Supplement: Supplementary file 1 — Supplementary material 1: Supplementary material includes Fig S1, S2, S3, S4, and S5. [file 13578_2024_1328_MOESM1_ESM.docx]

**Supplementary material**

**Enhancing striatal acetylcholine facilitates dopamine release and striatal output in parkinsonian mice**

Hongxia Li *et al.*

**Corresponding Authors:** Shengdi Chen, Email: [chensd@rjh.com.cn](mailto:chensd@rjh.com.cn); Ji Hu, Email: huji@shanghaitech.edu.cn

**The PDF file includes:** Figs. S1 to S5


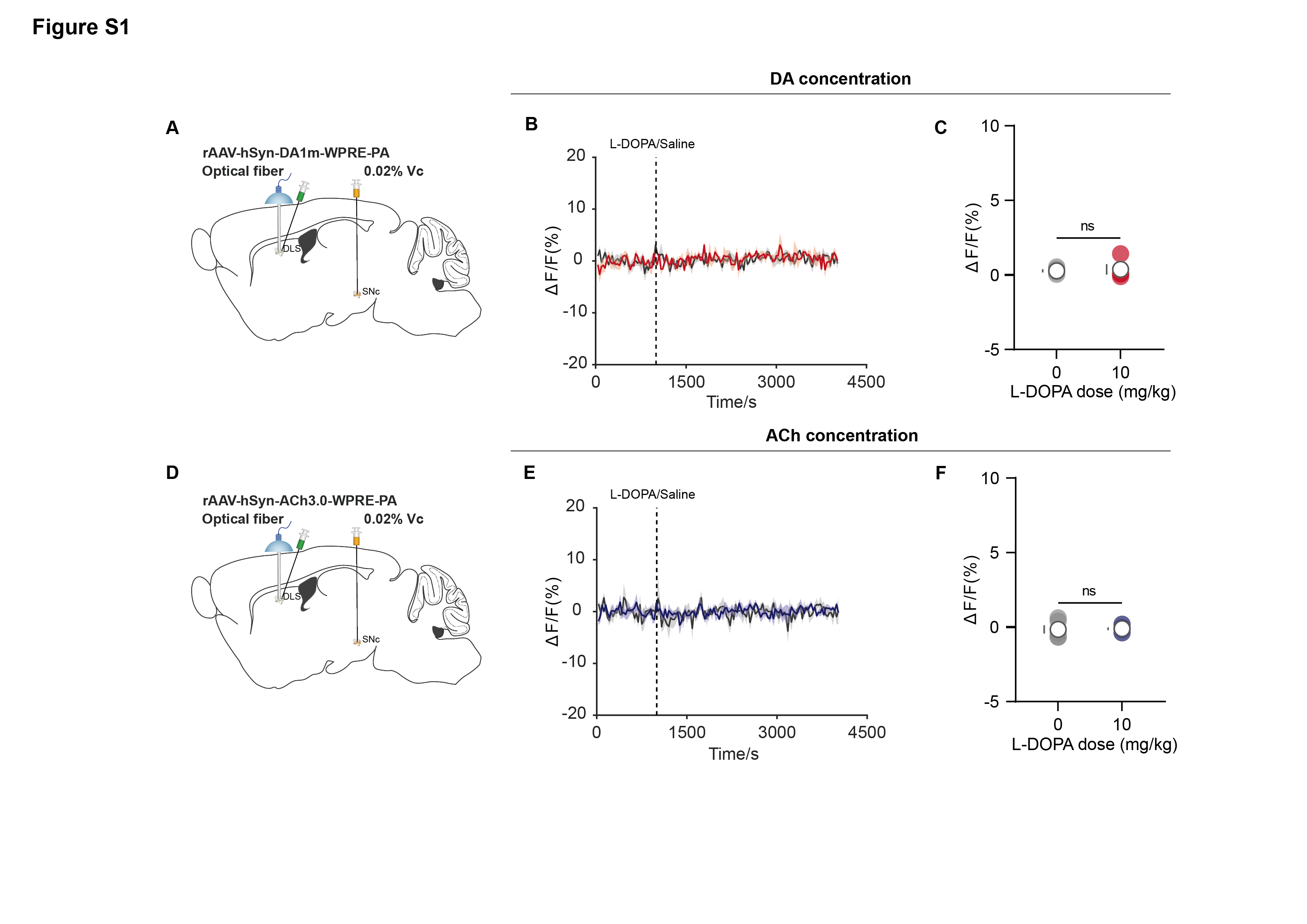


**Figure S1. Effect of high-dose L-DOPA on DLS DA and ACh concentration in sham-lesioned mice**

(A) Schematic diagram of the fiber photometry recording, 0.02% Vc injection into SNc, viral strategy for DA1m expression, and optical fiber implantation into DLS.

(B) DA1m sensor signals from DLS aligned to the moment of the administration of L-DOPA and saline. Mean values are shown as a red line (L-DOPA) and a black line (saline), SEM, interval is shaded in red and gray

(C) Quantification of change in DA1m sensor signals after administration of saline and L-DOPA [n=4, Two-tailed Paired t-test, t = 0.2077, df = 3, P = 0.8488].

(D) Schematic diagram of the fiber photometry recording, 0.02% Vc injection into SNc, viral strategy for ACh3.0 expression, and optical fiber implantation into DLS.

(E) ACh3.0 sensor signals from DLS aligned to the moment of the administration of L-DOPA and saline. Mean values are shown as a blue line (L-DOPA) and a black line (saline), SEM, interval is shaded in blue and gray

(F) Quantification of change in ACh3.0 sensor signals after administration of saline and L-DOPA [n = 5, Two-tailed Paired t-test, t = 0.2201, df = 4, P = 0.8366].

Data are presented as the mean ± SEM. *, p < 0.05; **, p < 0.01; ***, p < 0.001; ****, p < 0.0001; ns, not significant.

**
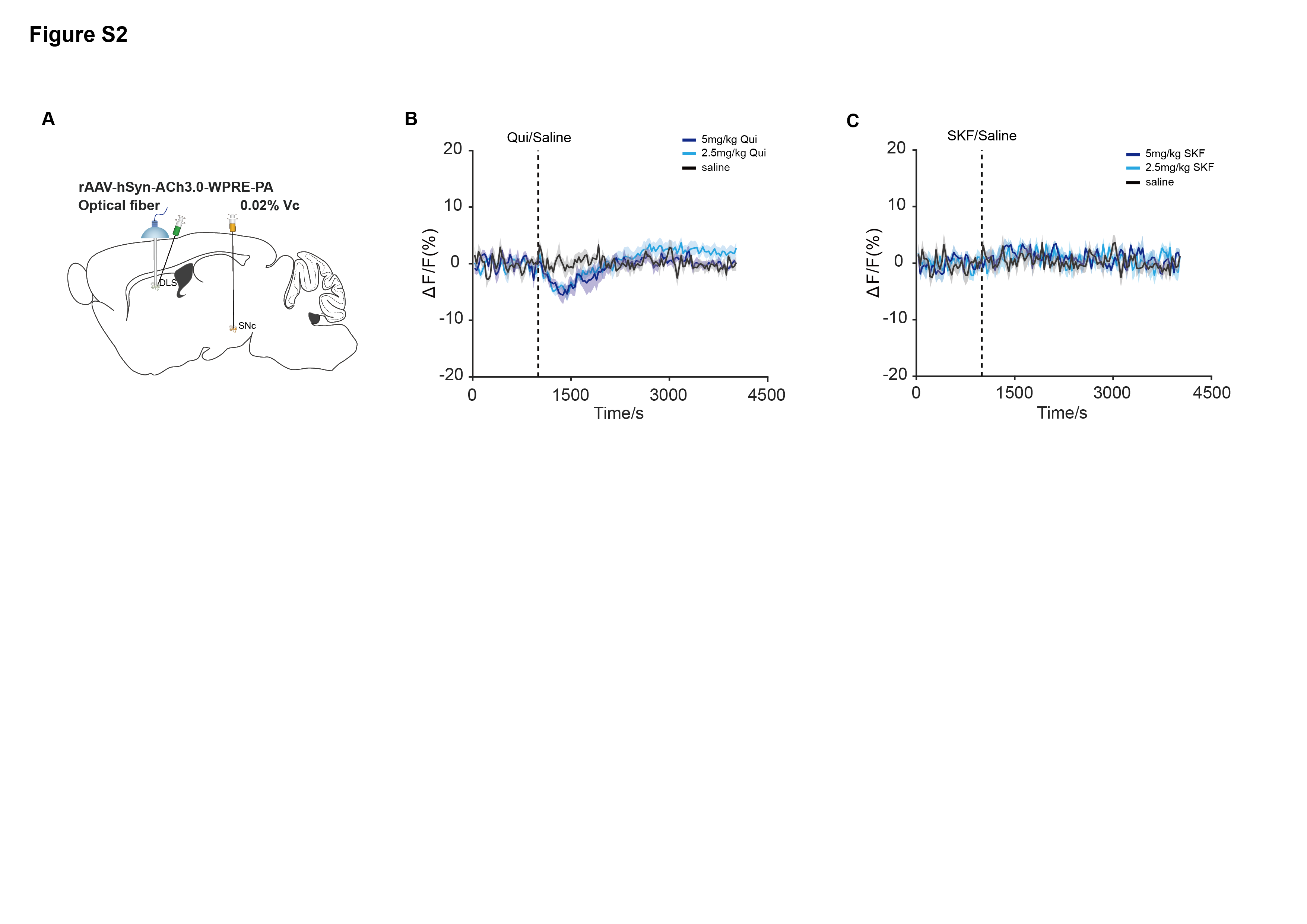
**

**Figure S2. Activation of D2R signaling regulated striatal ACh dynamics in sham-lesioned mice**

(A) Schematic diagram of the fiber photometry recording, 0.02% Vc injection into SNc, viral strategy for ACh3.0 expression, and optical fiber implantation into DLS.

(B and C) ACh3.0 sensor signals from DLS of sham-lesioned mice aligned to the moment of the administration of SKF, Qui, and saline. Mean values are shown as a blue line (SKF and Qui) and a black line (saline), SEM, interval is shaded in blue and gray.

**
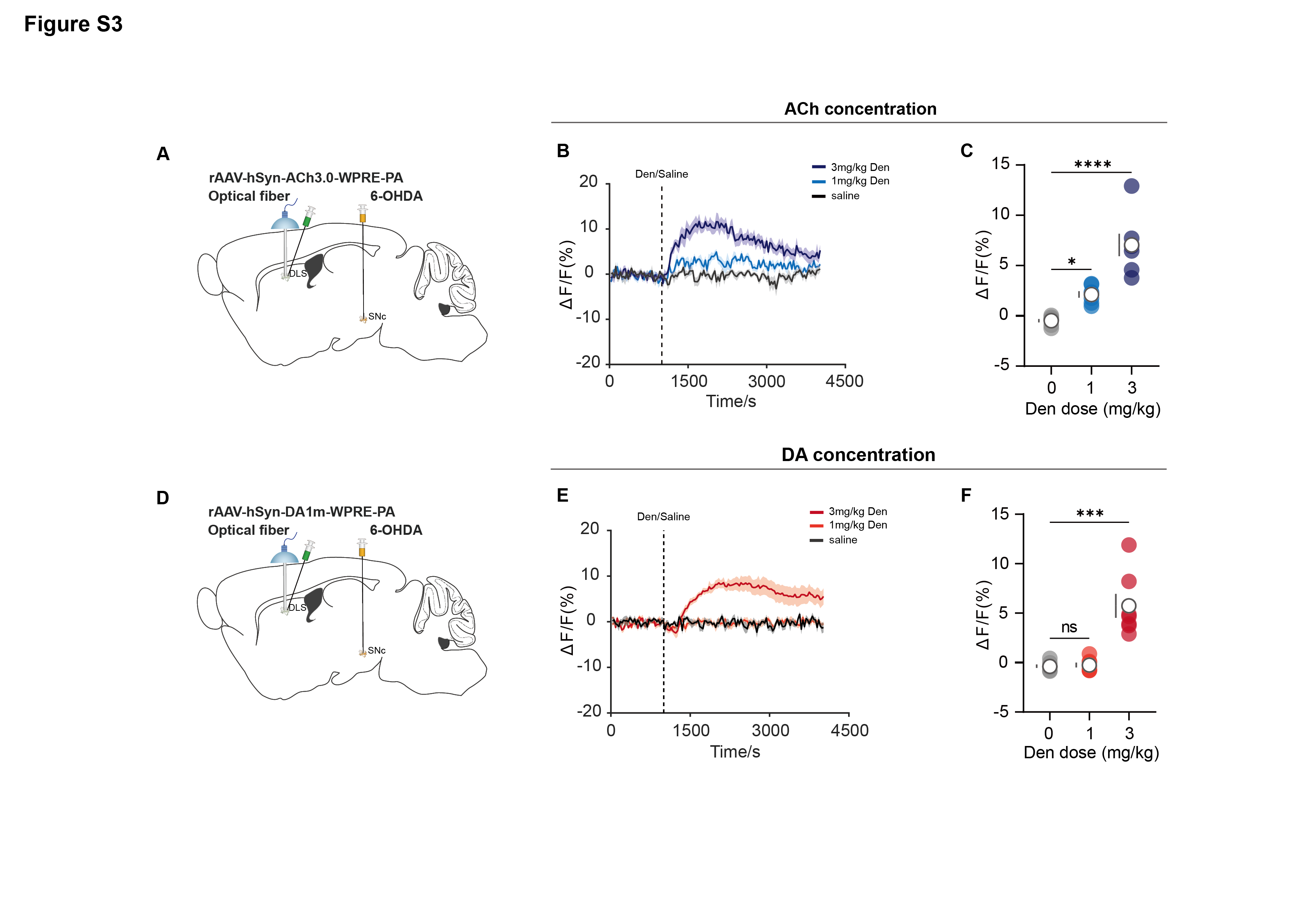
Figure S3. Effect of Donepezil on DLS ACh and DA concentration in parkinsonian mice**

(A) Schematic diagram of the fiber photometry recording, 6-OHDA injection into SNc, viral strategy for ACh3.0 expression, and optical fiber implantation into DLS.

(B) ACh3.0 sensor signals from DLS aligned to the moment of the administration of donepezil (Den) and saline. Mean values are shown as a blue line (Den) and a black line (saline), SEM, interval is shaded in blue and gray.

(C) Quantification of change in ACh3.0 sensor signals after administration of saline and Den [n = 7, F (2,12) = 29.46, P < 0.0001, RM one-way ANOVA with Post hoc Bonferroni’s test].

(D) Schematic diagram of the fiber photometry recording, 6-OHDA injection into SNc, viral strategy for DA1m expression, and optical fiber implantation into DLS.

(E) DA1m sensor signals from DLS aligned to the moment of the administration of Den and saline. Mean values are shown as a red line (Den) and a black line (saline), SEM, interval is shaded in red and gray.

(F) Quantification of change in DA1m sensor signals after administration of saline and Den [n = 7, F (2,12) = 22.20, P < 0.0001, RM one-way ANOVA with Post hoc Bonferroni’s test].


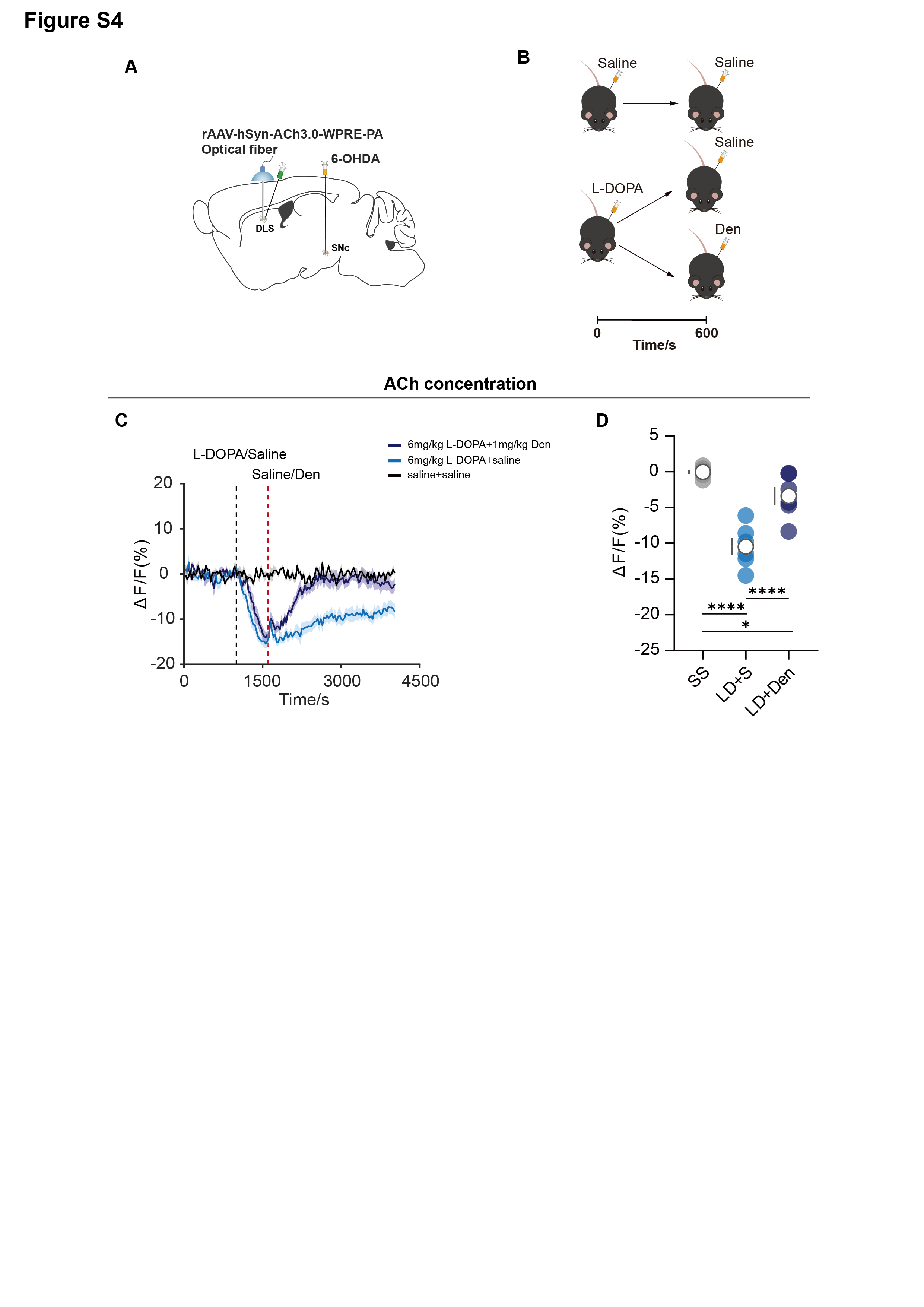


**Figure S4. Effect of low-dose Donepezil on DLS ACh level following L-DOPA administration in parkinsonian mice**

(A) Schematic diagram of the fiber photometry recording system, 6-OHDA injection into SNc, viral strategy for ACh3.0 expression, and optical fiber implantation into DLS.

(B) Schematic diagram of strategy for drugs application during the fiber photometry recording

(C) ACh3.0 sensor signals from DLS aligned to the moment of the administration of L-DOPA, donepezil (Den), and saline. Mean values are shown as a blue line (L-DOPA-saline and L-DOPA-Den) and a black line (saline-saline), SEM, interval is shaded in blue and gray.

(D) Quantification of change in ACh3.0 sensor signals after administration of saline and Den [n = 6, F (2,10) = 62.60, P < 0.0001, RM one-way ANOVA with Bonferroni’s post-hoc test].

**
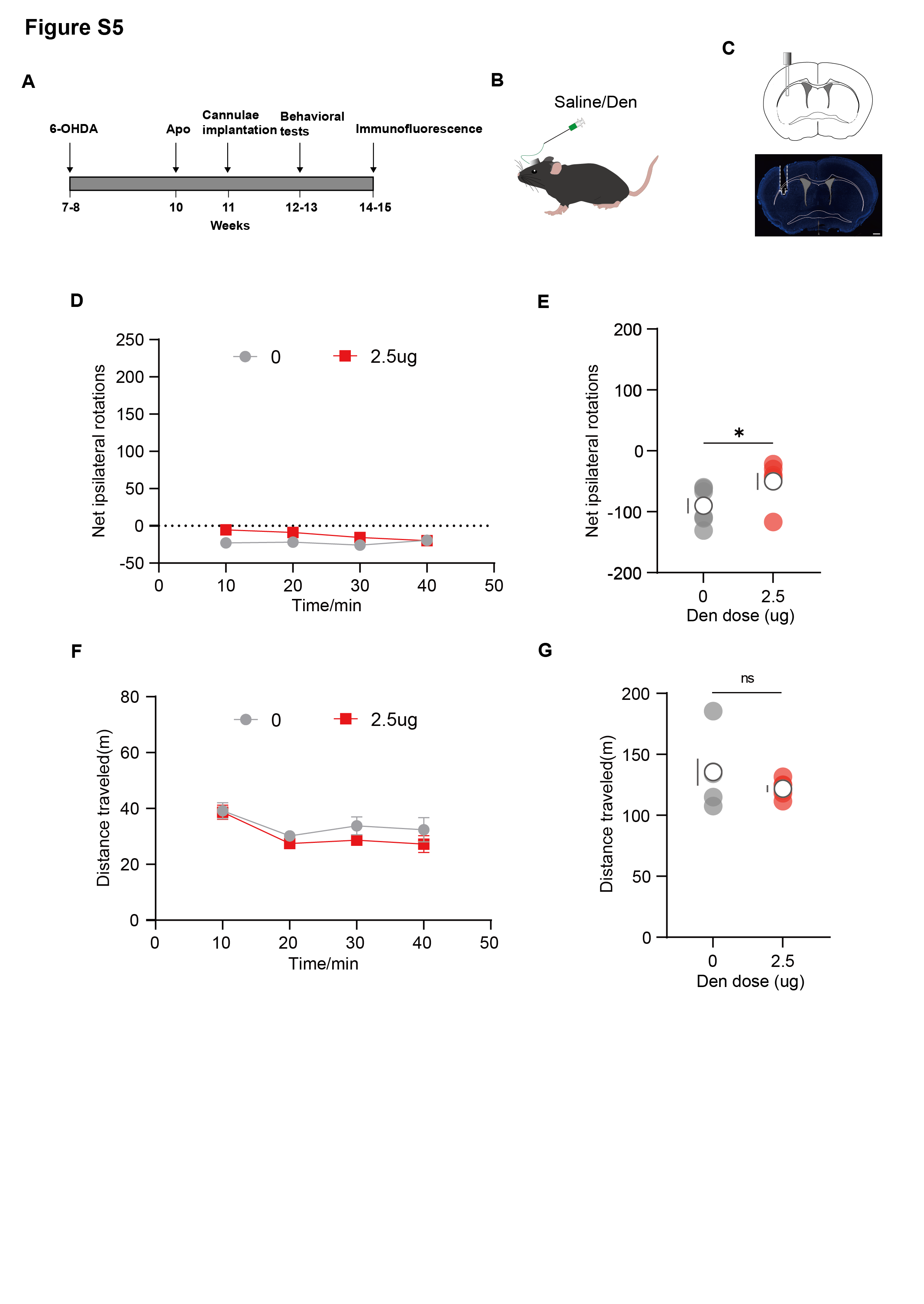
**

**Figure S5. Effects of enhancing DLS ACh on motor deficits in parkinsonian mice**

(A) Schematic drawing of experimental timeline.

(B) Schematic diagram of the strategy of drug injection for behavioral tests.

(C) Image of cannula channel in the coronal DLS section. Scale bar: 500um;

(D) Time trace showing effects of the different strategies of saline or 2.5ug Den injection on net ipsilateral rotations of mice, scored in 10-min time bins.

(E) Comparison of the net ipsilateral rotations in the first 40 min after administration of Den compared with saline [n = 6, Wilcoxon matched-pairs signed rank test, P = 0.0313].

(F) Time trace showing effects of the different strategies of saline or 2.5ug Den injection on total distance traveled of mice, scored in 10-min time bins.

(G) Comparison of the total distance traveled in the first 40 min after administration of Den compared with saline [n = 6, Wilcoxon matched-pairs signed rank test, P = 0.3125].
